# How Far Are We from Providing Pigs Appropriate Environmental Enrichment?

**DOI:** 10.3390/ani9100721

**Published:** 2019-09-24

**Authors:** Emma Fàbrega

**Affiliations:** Animal Welfare Programme, IRTA, Veïnat de Sies s/n, 17121 Monells, Catalonia, Spain; emma.fabrega@irta.cat

Limitations to the fulfilment of ethological and physiological needs can cause countless negative effects on animal welfare and lead to the development of abnormal behaviours. From a very young age, pigs are strongly motivated to perform exploratory and foraging behaviour, even if they are provided with enough feed to satisfy their dietary needs [1]. The widely used term “environmental enrichment” is usually understood as the addition of any element to an environment of captivity. However, from a scientific perspective, the concept of “environmental enrichment” refers to the improvement in the biological functioning of captive animals resulting from modifications to their environment [2]. To provide pigs with adequate enrichment, two main goals should be addressed: (1) the improvement of living conditions to allow the expression of species-specific behaviour, and (2) the development of strategies to manage undesirable behaviours, such as tail biting, and to prevent their escalation. 

Tail biting is a redirected behaviour that is said to be a response to insufficient stimulation and frustration in association with other negative environmental and management factors [3]. Although the exact triggering mechanisms remain unclear, tail biting has a multifactorial origin, and scientific evidence has identified a wide range of related environmental, dietary and husbandry risk factors. A lack of adequate enrichment material appears to be the initial risk factor that may trigger stress and has been considered a major cause for the most common type of tail biting, the so-called “two-stage tail biting”, which starts with gentle manipulation of another pig’s tail and proceeds to more intensive dental manipulation, bleeding and damage. Tail biting is not only an important welfare concern, but also has serious economic consequences for pig producers, because it lowers daily gains and increases susceptibility to secondary infections, antibiotic use and carcass condemnations.

Up to now, tail docking has been the most widely used preventive measure against tail biting adopted by farmers, although it is considered to cause acute pain and it does not totally prevent tail biting, since it does not address the underlying causes. EU legislation on pig welfare does not allow routine tail docking unless other measures such as environmental and management conditions have been tackled. Moreover, EU legislation specifically states that “pigs must have permanent access to a sufficient quantity of material to enable proper investigation and manipulation activities” [4]. However, the provision of adequate enrichment materials is not easy in modern intensive production systems, due to the prevalence of totally or partially slatted floors. There is a certain mismatch between materials that allow pigs to fulfil their behavioural needs (i.e., straw-type materials) and those not causing any blockage to the slurry systems (i.e., object-type materials). Therefore, an important challenge for researchers is to deliver data and practical solutions on enrichment materials that address pigs’ needs, that is, manipulable, investigable, safe, edible and chewable, but also suitable for implementation in intensive production systems. Moreover, most of the research on enrichment for pigs has focused on the prevention of tail biting, and therefore, there are gaps of knowledge on how to provide proper enrichment. While studies on the pre-partum period and materials for nest-building purposes have been completed [5], information about sows and piglets during lactation, boars or even gestating sows is scarcer.

Thus, the aim of this Special Issue was to collate recent research on “enrichment materials for pigs”, tailored to different climatic conditions and pig production systems.

This Special Issue contains 11 papers related to the enrichment of environments for pigs at different production stages (gestating sows, weaners and fattening pigs). The papers tackle the two previously mentioned main goals of enrichment. Many issues are highlighted in the contributions, and here we only mention a few.

The review paper by Van de Weerd and Ison [6] updates the question of how far we have come in enriching pig environments in the time since the same author (Van de Weerd) published a widely cited review ten years ago [7]. The paper compares the strategies adopted by the three main pig-producing regions (China, US and Western Europe), concluding that, although many improvements have been achieved, we are “still a long way off reaching the ultimate destination of an enriched pig population” (page 16) [6]. A commentary type paper discusses the effectiveness of legislation, especially within the EU context, on enhancing the use of proper enrichment materials to avoid routine tail docking.

In five of the papers, different enrichment materials for fattening pigs are compared, supporting interesting conclusions such as how the interest level of pigs is dependent on the characteristics, presentation, location and maintenance of the objects/materials. More destructible and chewable materials, such as straw in a rack or pieces of wood, were found to be more preferred than less manipulable materials. Tasks such as maintaining the objects’ cleanliness or adding new materials were found to be compatible with daily farming routines. One of the studies found a decrease in the interest of pigs over time, and suggested that elements of novelty should be investigated. In another study in which the type of enrichment was varied, tail damage was not reduced, but the decline in pigs’ interest towards enrichment was not as pronounced.

Two studies evaluated the effects of enrichment materials at the weaner stage and reported interesting findings, such as the fact that weaner pigs preferred olfactory to non-olfactory enrichment.

One study evaluated three different enrichment materials and presentations for gestating sows and confirmed that following changes in enrichment materials (with a rotation system, for example), sows showed an increased response to enrichment both at the group level and also in a sub-sample of dominant and subordinate focal sows.

One paper compares the effectiveness of relatively small amounts of straw on the floor, a rope or a Bite-Ride during tail-biting outbreaks as intervention measures to reduce the risk of an escalation in tail damage, finding a certain degree of reduction, but also suggesting the need of further research to find more efficient strategies.

Finally, the pig’s tail, its posture and/or movement, was used in some of the studies both to predict the likelihood of the occurrence of a tail-biting event (observing an increase in tucked tails), or as an indicator of positive emotional state (i.e., higher tail movement, as indicative of positive emotional state, was found in pigs when interacting with the enriched environment). This final study supports the definition of enrichment material as a way of improving biological functioning and welfare status (i.e., enrichment could enhance positive emotional state) resulting from the modification of a captive environment.
